# PER2 Promotes Odontoblastic/Osteogenic Differentiation of Dental Pulp Stem Cells by Modulating Mitochondrial Metabolism

**DOI:** 10.3390/ijms241310661

**Published:** 2023-06-26

**Authors:** Wushuang Huang, Qi Huang, Hongwen He, Fang Huang

**Affiliations:** 1 Hospital of Stomatology, Sun Yat-sen University, Guangzhou 510055, China; huangwsh26@mail.sysu.edu.cn (W.H.);; 2 Guangdong Provincial Key Laboratory of Stomatology, Guangzhou 510055, China; 3 Institute of Stomatology, Sun Yat-sen University, Guangzhou 510055, China

**Keywords:** PER2, dental pulp stem cells, odontoblastic/osteogenic differentiation, mitochondrial metabolism

## Abstract

Human dental pulp stem cells (hDPSCs) possess remarkable self-renewal and multilineage differentiation ability. PER2, an essential circadian molecule, regulates various physiological processes. Evidence suggests that circadian rhythm and PER2 participate in physiological functions of DPSCs. However, the influence of PER2 on DPSCs’ differentiation remains largely unknown. This study aimed to explore the effect and potential mechanism of PER2 on hDPSCs’ differentiation. Dental pulp tissues were extracted, and hDPSCs were cultured for *in vitro* and *in vivo* experiments. Dorsal subcutaneous transplantation was performed in 6-week-old male BALB/c mice. The hDPSCs’ odontoblastic/osteogenic differentiation was assessed, and mitochondrial metabolism was evaluated. The results indicated PER2 expression increasing during hDPSCs’ odontoblastic/osteogenic differentiation. Gain- and loss-of function studies confirmed that PER2 promoted alkaline phosphatase (ALP) activity, mineralized nodules deposition, mRNA expression of *DSPP*, *DMP1*, *COL1A1* and protein expression of DSPP and DMP1 in hDPSCs. Furthermore, PER2 enhanced collagen deposition, osteodentine-like tissue formation and DSPP expression *in vivo*. Mitochondrial metabolic evaluation aimed to investigate the mechanism of PER2-mediated hDPSC odontoblastic/osteogenic differentiation, which showed that PER2 increased ATP synthesis, elevated mitochondrial membrane potential and changed expression of proteins regulating mitochondrial dynamics. This study demonstrated that PER2 promoted hDPSCs’ odontoblastic/osteogenic differentiation, which involved mitochondrial metabolic change.

## 1. Introduction

Human dental pulp stem cells (hDPSCs) are ectodermal-derived stem cells, originating from neural crest cells and possessing properties of mesenchymal stem cells. Human DPSCs have the ability to differentiate into odontoblast-like cells, osteoblast, neural-like cells, adipocytes and chondrocytes under specific stimuli [1]. In comparison to other adult stem cells, such as bone marrow mesenchymal stem cells (BMMSCs), hDPSCs exhibit a higher proliferation rate, and increased clonogenic and mineralization potential [2]. However, the molecular mechanisms underlying the odontoblastic/osteogenic differentiation of hDPSCs have not been fully elucidated.

The circadian clock synchronizes the behavior and physiology of all living organisms with the cyclic changes of the external environment. Increasing evidence suggests that the circadian clock influences oral health and tooth development [3,4,5,6,7]. Circadian molecules, such as BMAL1, PER2, PER1, and CLOCK, have exhibited fluctuating expression in murine dental pulp cells at various stages of development [4]. Furthermore, temporal clock genes have been observed to oscillate in DPSCs in response to rhythmic mechanical stretch [8]. Additionally, the circadian rhythm of dental pulp sensibility in humans undergoes changes with age, and exhibits significant differences among diabetic patients, hypertensive patients, and healthy individuals [5,6,7]. These findings indicate the involvement of circadian rhythm in the physiological and pathological processes of dental pulp cells.

PER2, as a core component of the molecular clock network, is fundamental for organ development, stem cell differentiation and tissue restoration. Previous studies have demonstrated that PER2 regulates bone volume, bone formation rate, and osteogenic differentiation of BMMSCs in mice [9,10,11]. Dentin and bone, both being hard tissues in the body, share many similarities in terms of their composition and formation, including genes and signaling pathways involved in regulating odontoblastic and osteogenic differentiation. While both BMMSCs and DPSCs possess the potential for odontoblastic/osteogenic differentiation [12,13,14], as mentioned above, DPSCs have distinct advantages. In carious teeth, the transcription level of *PER2* was downregulated in dental pulp [15]. Moreover, in a previous study, we observed that PER2 expression was downregulated in dental pulp cells of mice under circadian disrupted conditions [16]. Additionally, the mRNA expression of *PER2* changed in DPSCs responding to LPS stimulation [17]. Taken together, the author hypothesized that PER2 might be involved in the physiological functions of dental pulp stem cells, potentially influencing the process of cell differentiation.

Mitochondria play a crucial role in cellular energy metabolism and homeostasis, exerting significant influence on cell fate determination and bioactivities [18,19]. Accumulating evidence suggests that mitochondria play a role in determining the fate and differentiation of stem cells through multiple functions, including ATP generation, mitochondrial dynamics, and pyruvate metabolism [19]. The behaviors and functions of mitochondria, such as changes in membrane potential, ATP generation, ROS emission activity, and Ca^2+^ regulation, have been implicated in the differentiation process of dental pulp-derived stem cells [20,21]. Furthermore, circadian oscillations have been detected not only in mitochondrial gene expression but also in key mitochondrial bioenergetic parameters, such as mitochondrial membrane potential (MMP) and the activity of mitochondrial enzymes [22,23]. PER1/2 depletion attenuated the diurnal regulation of mitochondrial respiration in mice, and rate-limiting mitochondrial enzymes involved in the accumulation of lipids and carbohydrates were found to be dependent on PER1/2 [24]. Based on the aforementioned findings, we propose that PER2 may regulate DPSCs’ differentiation by influencing mitochondrial metabolism.

The aim of this study is to investigate the influence of PER2 on the odontoblastic/osteogenic differentiation of hDPSCs and explore the potential correlation between PER2 and the mitochondrial metabolic state during this process. Our study revealed that PER2 enhanced hDPSCs odontoblastic/osteogenic differentiation, resulting in osteodentine-like tissue formation and increased collagen deposition. Moreover, our results indicated that mitochondrial metabolism was involved in the PER2-regulated odontoblastic/osteogenic differentiation process of hDPSCs.

## 2. Results

### 2.1. The Expression Pattern of PER2 in Human Dental Pulp Tissue

**Figure 1:**
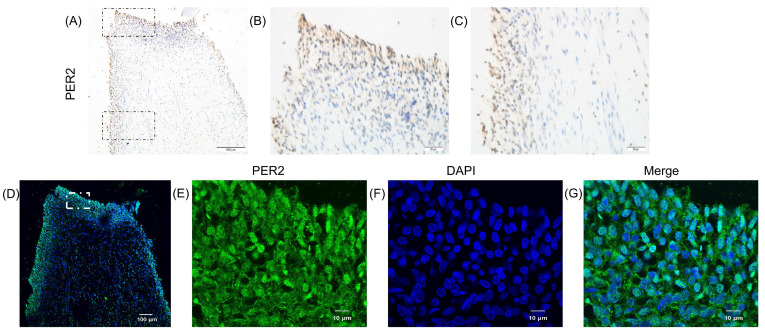
PER2 expression pattern in human dental pulp tissue. Dental pulp tissues were obtained from human third molars and subjected to fixation, dehydration, embedding, sectioning and immunohistochemistry (IHC) and immunofluorescence (IF) analyses. (**A**–**C**) Cells at the pulp periphery adjacent to dentin exhibited higher levels of PER2 expression compared to cells in the pulp core. In panel (**A**) the upper and lower regions enclosed by black dotted squares represent areas where higher resolution images (40×) were captured for the coronal pulp (**B**) and the pulp near the radicular zone (**C**), respectively. (**D**–**G**) PER2-positive staining was observed in the cytoplasm and nucleus of cells at the pulp periphery adjacent to dentin, displaying stronger staining intensity compared to cells in the pulp core (**D**). Magnified regions (100×) within the white dotted square in (**D**) are presented in (**E**–**G**). Scale bar: (**A**,**D**) 100 μm; (**B**,**C**) 20 μm; (**E**–**G**) 10 μm.

To investigate the expression pattern of PER2 in human dental pulp, the dental pulp tissues were extracted from human third molars and processed for immunohistochemistry and immunofluorescence. PER2 expression levels were higher in cells at the pulp periphery adjacent to dentin compared to those in the pulp core (Figure 1A–D). Furthermore, PER2-positive staining was observed in both the cytoplasm and nucleus (Figure 1E–G).

### 2.2. Characterization and Multi-Differentiation Capacity of hDPSCs

**Figure 2:**
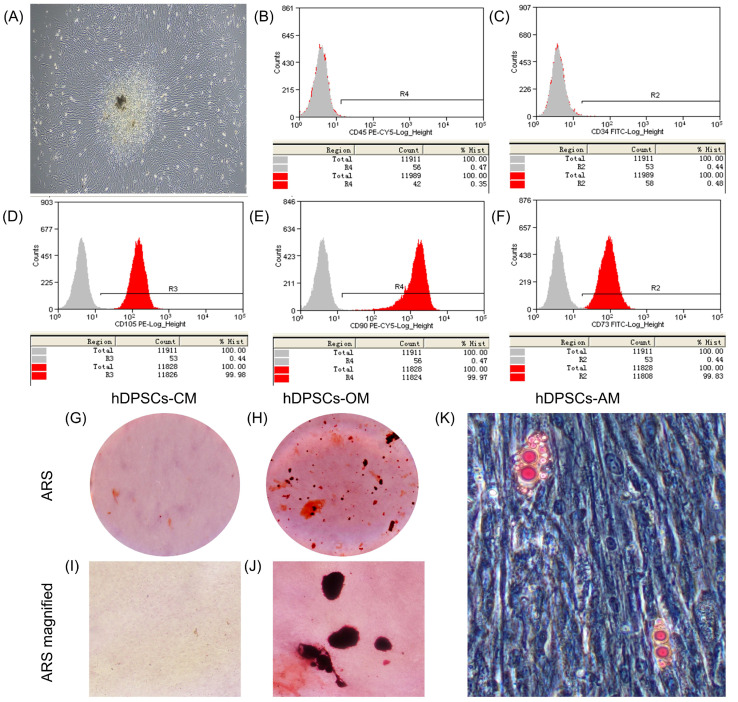
The isolation, culture and identification of hDPSCs. (**A**) Primary hDPSCs were isolated and cultured for 10 days, displaying a spindle-shaped and fibroblastic-like morphology (4×). (**B**–**F**) Immunophenotype analysis of hDPSCs by flow cytometry showed positive expression of CD105 (**D**), CD90 (**E**), CD73 (**F**), with negative expression of CD45 (**B**) and CD34 (**C**). (**G**–**K**) Alizarin Red S staining revealed that hDPSCs cultured in CM exhibited minimal formation of mineralized nodules (**G**,**I**), whereas those cultured in OM showed evident mineralized nodules (**H**,**J**). The Oil Red O staining of hDPSCs after 19 days of adipogenic induction demonstrated the presence of lipid droplets (20×) (**K**). (**I**) and (**J**) are magnified regions of (**G**) and (**H**), respectively. hDPSCs, human dental pulp stem cells; CM, control medium; OM, odontoblastic/osteogenic medium; AM, adipogenic medium; ARS, Alizarin Red S.

Human DPSCs were isolated and cultured from healthy dental pulp. The morphology of hDPSCs exhibited spindle-shaped and fibroblastic-like appearance (Figure 2A). Flow cytometry analysis confirmed the expression of specific surface markers CD73, CD90, and CD105 on hDPSCs, while they were negative for CD34 and CD45 (Figure 2B–F). Furthermore, when hDPSCs were cultured in specific differentiation media, they displayed the ability to undergo osteogenic and adipogenic differentiation, as observed by the formation of mineralized nodules and lipid droplets, respectively (Figure 2G–K). These findings indicate the multidirectional differentiation potential of hDPSCs.

### 2.3. Effect of Odontoblastic/Osteogenic Induction on PER2 Expression in hDPSCs

**Figure 3:**
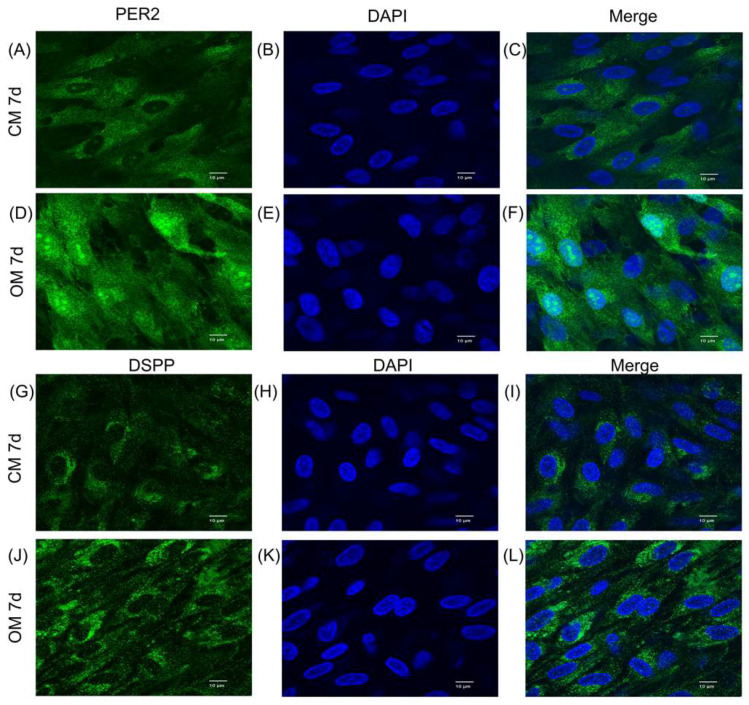
The promotion of PER2 expression was observed during odontoblastic/osteogenic differentiation of hDPSCs. (**A**–**L**) hDPSCs were cultured in either CM (**A**–**C**,**G**–**I**) or OM (**D**–**F**,**J**–**L**) for 7 days. Immunofluorescence staining was performed to examine the expression of PER2 (**A**–**F**) and DSPP (**G**–**L**). The results showed a dramatic increase in both PER2 and DSPP expression in cells cultured in OM. PER2 exhibited nuclear and cytoplasmic localization in OM-cultured DPSCs (**D**–**F**), while it was predominantly localized in the cytoplasm in CM-cultured cells (**A**–**C**). hDPSCs, human dental pulp stem cells; CM, control medium; OM, odontoblastic/osteogenic medium. Scale bar: (**A**–**L**) 10 μm.

To investigate the impact of differentiation induction on PER2 expression in hDPSCs, the cells were cultured in either control medium (CM) or odontoblastic/osteogenic medium (OM) and harvested on day 7. Compared to cells cultured in CM (Figure 3A–C,G–I), hDPSCs cultured in OM exhibited a dramatic increase in PER2 and DSPP immunofluorescence staining (Figure 3D–F,J–L). Additionally, PER2 was observed to localize in both the nucleus and cytoplasm of OM-cultured hDPSCs (Figure 3D–F), whereas in CM-cultured cells, it predominantly resided in the cytoplasm (Figure 3A–C).

### 2.4. PER2 Enhances Odontoblastic/Osteogenic Differentiation Potential of hDPSCs

To further investigate the effect of PER2 on hDPSCs differentiation, *PER2*-knockdown with lentiviral vectors *PER2*-sh1 and *PER2*-sh2 and *PER2*-overexpression (*PER2*-OE) hDPSCs were constructed. The efficiency of *PER2* knockdown and overexpression in hDPSCs was validated by qRT-PCR and Western blot analysis (Figure S1A–C,E–G). As shown in figure (Figure S1B,C), the protein expression level of PER2 was significantly downregulated in *PER2*-sh2 cells while slightly decreased in *PER2*-sh1 cells. Therefore, *PER2*-sh2 cells were selected for further experiments.

Cell proliferation rate of *PER2*-sh2 and the control hDPSCs show no significant difference when cultured in CM for 1 day and 3 days. However, after 5 days and 8 days of culture in CM, the cell proliferation rate was slightly higher in *PER2*-sh2 hDPSCs compared to the control group (Figure S1D). Cell proliferation rates of *PER2*-OE and the control hDPSCs did not show a significant difference when cultured in CM for 1 day, 3 days, 5 days and 8 days (Figure S1H).

**Figure 4:**
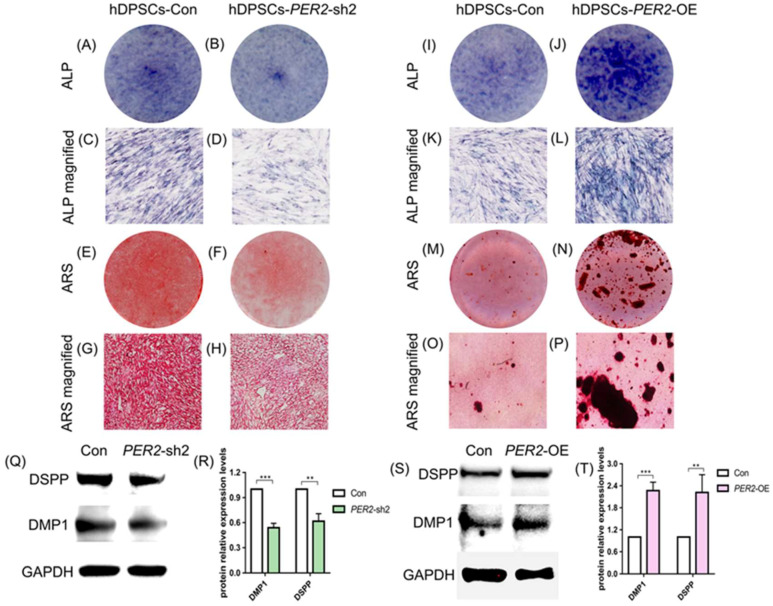
PER2 enhances the odontoblastic/osteogenic differentiation potential of hDPSCs. (**A**–**D**) Cells were cultured in OM for 7 days and then harvested. ALP staining revealed a significant decrease in ALP activity in *PER2*-sh2 hDPSCs (**B**,**D**) compared to the control group (**A**,**C**). (**E**–**H**) Cells were cultured in OM for 21 days and harvested. Alizarin Red S staining presented fewer mineralized nodules in *PER2*-sh2 hDPSCs (**F**,**H**) compared to the control group (**E**,**G**). (**I**–**L**) Cells were cultured in OM for 7 days and harvested. Compared to the control group (**I**,**K**), *PER2*-overexpression upregulated ALP activity in hDPSCs (**J**,**L**). (**M**–**P**) Cells were cultured in OM for 21 days and harvested. Compared to the control group (**M**,**O**), Alizarin Red S staining presented more mineralized nodules in *PER2*-overexpression hDPSCs (**N**,**P**). (**C**,**D**,**G**,**H**,**K**,**L**,**O**,**P**) are magnified images of (**A**,**B**,**E**,**F**,**I**,**J**,**M**,**N**), respectively. (**Q**,**R**) Cells were cultured in OM for 7 days and harvested. Western blot and quantitative analysis of odontoblastic differentiation related proteins DSPP and DMP1 in *PER2-*knockdown hDPSCs. *PER2* depletion led to reduced expression of DSPP and DMP1. (**S**,**T**) Cells were cultured in OM for 7 days and harvested. Western blot and quantitative analysis of DSPP and DMP1 in *PER2-*overexpression hDPSCs. Overexpression of *PER2* elevated the expression of DSPP and DMP1 hDPSCs, human dental pulp stem cells; ALP, alkaline phosphatase. Data are presented as mean ± SEM. ** *p* < 0.01, *** *p* < 0.001.

To assess the effect of PER2 on hDPSCs’ differentiation, ALP staining, Alizarin Red S staining and detection of differentiation markers were performed. ALP staining revealed a significant decrease in ALP activity, and Alizarin Red S staining presented fewer mineralized nodules in *PER2*-sh2 hDPSCs compared to the control group (Figure 4A–H). *PER2* depletion resulted in reduced mRNA expression of *DMP1*, *DSPP*, *ALP*, and *COL1A1* in hDPSCs (Figure S2A), as well as downregulated expression of dentinogenic differentiation-related proteins DSPP and DMP1 (Figure 4Q,R). Conversely, *PER2*-overexpression upregulated ALP activity (Figure 4I–L), mRNA expression of *DMP1*, *DSPP*, *COL1A1* (Figure S2B) and protein expression level of DSPP and DMP1 (Figure 4S,T) in hDPSCs. Alizarin Red S staining also presented an increased number of mineralized nodules in *PER2*-overexpression hDPSCs (Figure 4M–P).

### 2.5. Overexpression of PER2 in hDPSCs Enhances Collagen Secretion and Osteodentine-like Structure Formation

**Figure 5:**
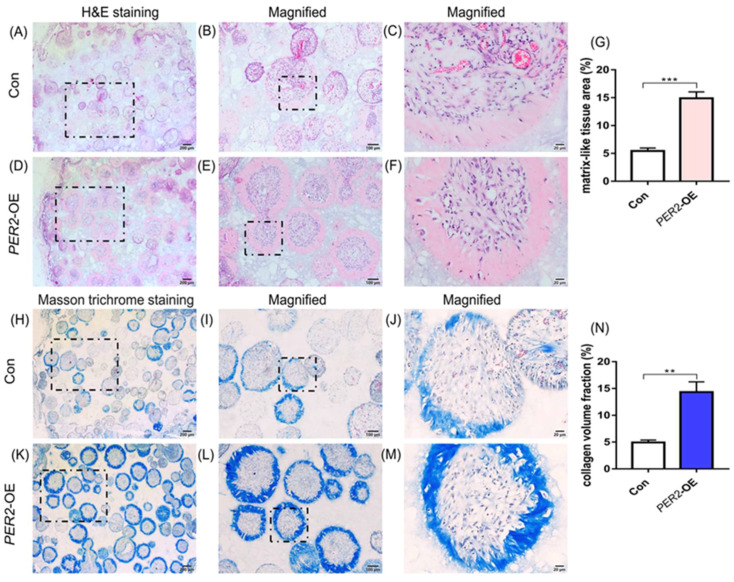
Overexpression of *PER2* in hDPSCs enhances collagen deposition and osteodentine-like structure formation. β-TCP scaffolds were loaded with hDPSCs and transplanted into 6-week-old male BALB/c nude mice subcutaneously for 8 weeks. Samples were fixed, demineralized, dehydrated, embedded, sectioned. (**A**–**G**) H&E staining showed that hDPSCs colonized in the pores of the scaffolds with the presence of loose connective tissue and osteodentine-like structure was formed around the outer-layer of the cells. Compared with the control group (**A**–**C**), the *PER2*-overexpression group exhibited increased mineralized osteodentine-like formation (**D**–**F**). Quantitative analysis of osteodentine-like tissue area was performed by Image-Pro Plus 6.0 (**G**). (**H**–**N**) Compared to the control group (**H**–**J**), the *PER2*-overexpression group exhibited increased collagen deposition according to Masson trichrome staining (**K**–**M**). Quantitative analysis of collagen volume area was conducted (**N**). (**B**,**E**,**I**,**L**) represented the magnified regions (10×) in (**A**,**D**,**H**,**K**) black dotted squares, respectively. (**C**,**F**,**J**,**M**) represent the magnified regions (40×) in (**B**,**E**,**I**,**L**) black dotted squares, respectively. Scale bar: (**A**,**D**,**H**,**K**) 200 μm; (**B**,**E**,**I**,**L**) 100 μm; (**C**,**F**,**J**,**M**) 20 μm. Data are presented as mean ± SEM. ** *p* < 0.01, *** *p* < 0.001.

**Figure 6:**
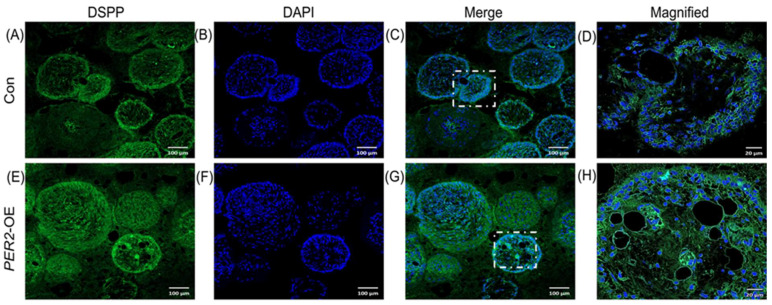
Overexpression of *PER2* in hDPSCs enhances DSPP expression *in vivo*. β-TCP scaffolds were loaded with hDPSCs and transplanted into 6-week-old male BALB/c nude mice subcutaneously for 8 weeks. Samples were fixed, demineralized, dehydrated, embedded, sectioned. (**A**–**H**) Immunofluorescence staining of DSPP, an odontoblastic maker, revealed cytoplasmic staining in cells, with stronger staining observed in the *PER2*-overexpression group (**E**–**H**) compared to the control group (**A**–**D**). (**D**,**H**) represented the magnified regions (40×) in (**C**,**G**) white dotted squares, respectively. Scale bar: (**A**–**C**,**E**–**G**) 100 μm; (**D**,**H**) 20 μm.

To confirm the impact of PER2 on the odontoblastic/osteogenic differentiation of hDPSCs *in vivo*, β-TCP scaffolds were loaded with hDPSCs and transplanted subcutaneously into BALB/c nude mice for 8 weeks. All surgical sites healed successfully without any signs of infection or scaffold exposure. HE staining showed the colonization of hDPSCs within the scaffold pores, with the presence of loose connective tissue and the formation of osteodentine-like structures around the outer layer of the cells (Figure 5A–F). Additionally, HE and Masson trichrome staining showed the presence of entrapped cells within the osteodentine-like tissue and collagen within the hDPSCs-β-TCP scaffolds (Figure 5A–F,H–M). Compared to the control group (Figure 5A–C,H–J), the *PER2*-overexpression group exhibited increased osteodentine-like tissue formation (Figure 5D–F) and collagen deposition (Figure 5K–M), as confirmed by HE and Masson trichrome staining. Immunofluorescence staining of DSPP, an odontoblastic maker, revealed cytoplasmic expression in the cells (Figure 6A–H), with stronger staining observed in the *PER2*-overexpression group (Figure 6E–H) compared to the control group (Figure 6A–D).

### 2.6. Mitochondrial Metabolism Evaluation in PER2-Knockdown or Overexpression hDPSCs during the Odontoblastic/Osteogenic Differentiation Process

In order to explore the potential mechanism underlying the PER2-regulated odontoblastic/osteogenic differentiation of DPSCs, we evaluated DPSCs’ mitochondrial functions by monitoring ATP synthesis, mitochondrial activities through measuring mitochondrial membrane potential (MMP), and mitochondrial behaviors via assessing fusion and fission dynamics using Western blot analysis.

ATP synthesis serves as an indicator of mitochondrial function [25]. Firstly, hDPSCs were cultured in either CM or OM and harvested on days 1, 3, and 7. Compared to the cells cultured in CM, hDPSCs incubated in OM showed a higher intracellular ATP level. However, there was no significant difference in ATP generation among the three groups of hDPSCs cultured in OM (Figure S3A). The intracellular ATP levels were lower in the *PER2*-sh2 hDPSCs compared to the control group, regardless of whether they were cultured in CM or OM (Figure S3B). Similarly, overexpression of *PER2* increased intracellular ATP generation (Figure S3C). 

**Figure 7:**
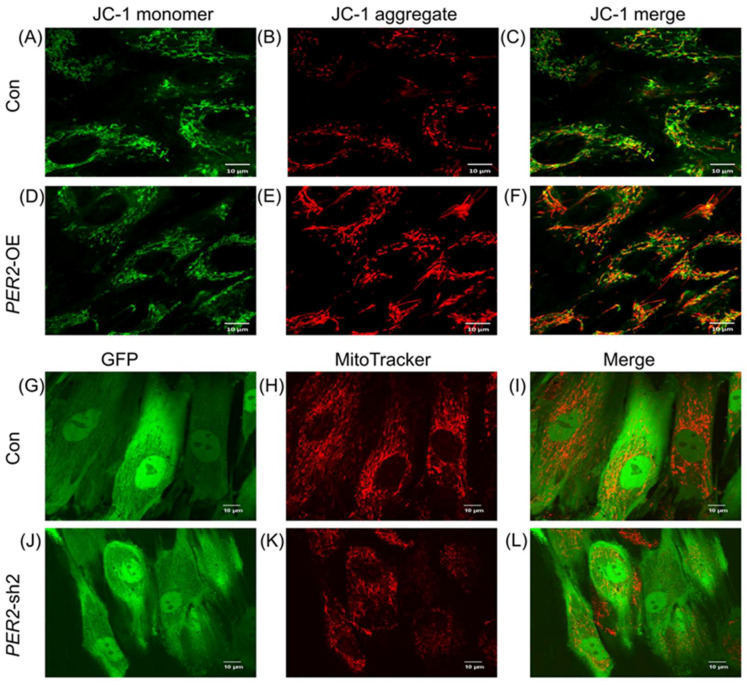
Mitochondrial membrane potential evaluation in *PER2*-knockdown and *PER2*-overexpression hDPSCs. (**A**–**F**) A higher number of JC-1 aggregates in *PER2*-overexpression cells (**D**–**F**) compared to the control cells (**A**–**C**). (**G**–**L**) Cells transfected with lentivirus vectors of *PER2*-sh2 and the control one showed fluorescence upon GFP excitation. Compared to the control group (**G**–**I**), *PER2* depletion resulted in reduced Mitotracker Red CMXRos staining in hDPSCs (**J**–**L**). Scale bar: (**A**–**L**) 10 μm.

The MMP serves as the direct chemical driving force for ATP generation in cells and exerts vital effects for various mitochondrial functions and behaviors [25]. To investigate the effect of PER2 on MMP during the odontoblastic/osteogenic differentiation process of hDPSCs, the JC-1 or Mitotracker Red CMXRos dye was used. JC-1 dye exhibits potential-dependent accumulation in mitochondria by a fluorescence emission shift from green (representing JC-1 monomer) to red (representing JC-1 aggregates). A higher number of JC-1 aggregates were observed in the *PER2*-OE cells (Figure 7D–F) compared to the control cells, indicating an enhanced MMP (Figure 7A–C). Mitotracker Red CMXRos stains mitochondria in live cells, and its accumulation depends on the membrane potential. The lentivirus vectors of *PER2*-sh2 and the control vector were inserted with the *EGFP* sequence, enabling cells to fluoresce upon GFP excitation (Figure 7G,J). Compared to the control group (Figure 7H), *PER2* depletion resulted in reduced Mitotracker Red CMXRos staining in hDPSCs (Figure 7K).

**Figure 8:**
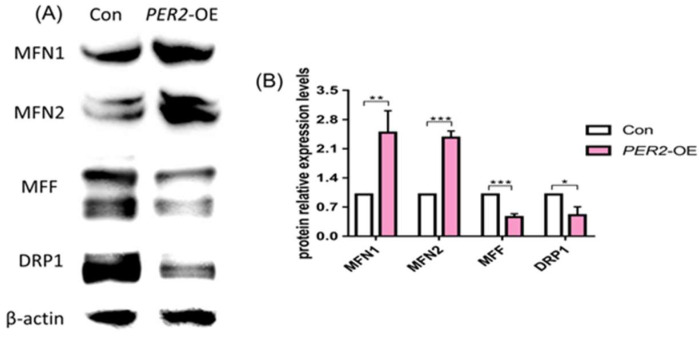
Detection of proteins that regulate mito-fisson and mito-fusion in *PER2*-overexpression hDPSCs. (**A**,**B**) *PER2*-overexpression upregulated the expression level of MFN1 and MFN2 while reducing MFF and DRP1 expression in hDPSCs at a level of statistical significance. Data are presented as mean ± SEM. * *p* < 0.05, ** *p* < 0.01, *** *p* < 0.001.

Additionally, proteins regulating mitochondrial fission and fusion were detected. *PER2*-overexpression upregulated the expression level of mitofusin 1 (MFN1) and mitofusin 2 (MFN2) while reducing the expression of the mitochondrial fission factor (MFF) and dynamin-related protein 1 (DRP1) in hDPSCs with statistical significance (Figure 8A,B).

## 3. Discussion

Over the past decade, hDPSCs isolated from dental pulp tissues have obtained extensive attention in the fields of tissue engineering and regenerative medicine, primarily due to their accessibility and multilineage differentiation capacity [26]. Research interest in DPSCs has increased significantly, given their potential for various clinical applications in dental and other tissue regeneration, as well as in disease treatment [26,27]. While the involvement of circadian rhythm and clock genes in the physiological and pathological processes of dental pulp and DPSCs has been established [5,6,7,8,17], their relevance to the odontoblastic/osteogenic differentiation of DPSCs still needs to be elucidated. In this study, we identified PER2, a critical circadian molecule, as a regulator of odontoblastic/osteogenic differentiation of hDPSCs by inducing changes in mitochondrial metabolism.

The omnipresent circadian rhythm permeates the physiological and pathophysiology process. Epidemiological studies have shown that individuals with long-term irregular lifestyles have a higher risk of inflammation, metabolic disorders, immune system imbalances, cancer and cardiovascular diseases, which are increasingly prevalent in contemporary society [28,29]. Based on our previous study and the aforementioned evidence, it is speculated that PER2 may play a role in the physiological functions of dental pulp cells under the influence of the circadian rhythm [16]. In this study, we first examined the expression pattern of PER2 in human dental pulp and during odontoblastic/osteogenic differentiation induction. PER2 was highly expressed in the cells at the periphery of the pulp adjacent to the dentin, and its expression dramatically increased when hDPSCs were cultured in odontoblastic/osteogenic medium *in vitro*. These results suggested that the expression level of PER2 may be related to the differentiation process of hDPSCs.

Next, we successfully generated *PER2*-knockdown and *PER2*-overexpression hDPSCs and detected their function. The proliferation of hDPSCs was not significantly affected by PER2. Gain- and loss-of-function studies revealed that PER2 increased the expression level of *COL1A1* and odontoblastic differentiation markers DSPP and DMP1, upregulated ALP activity, and enhanced the deposition of mineralized nodules. ALP activity is closely associated with the production of mineralized tissue [30]. COL1A1 supramolecularly assembles as type Ⅰ collagen, which is the most abundant organic material in mature dentin [31]. Alizarin Red S staining indicated mineralization or calcification, a late hallmark for osteogenic differentiation [32]. Collectively, our *in vitro* experiments provided evidence that PER2 played an important role in the odontoblastic/osteogenic differentiation potential of hDPSCs.

Furthermore, in the *PER2*-overexpression hDPSCs-β-TCP scaffolds, we observed the formation of mineralized osteodentine-like structures and abundant collagen deposition, accompanied by significantly stronger DSPP immunofluorescence staining. These findings proved that PER2 exerted a superior mineralization potential by influencing the odontoblastic/osteogenic differentiation of hDPSCs. Additionally, we noted the presence of osteocyte lacunae-like structures with entrapped cells in hDPSCs-β-TCP scaffolds. Given that hDPSCs possess osteogenic potential and the involvement of PER2 in bone homeostasis and regeneration has been reported previously [10,11,33], it is plausible that PER2 also plays a role in the osteogenic differentiation of hDPSCs *in vivo*. However, further investigations are required to validate this hypothesis, as detailed experiments are currently lacking. Recent studies have demonstrated that bone formation and bone turnover markers exhibit circadian rhythms [34,35]. Additionally, *Per2* knockout or mutant mice have showed elevated mineral apposition rate and bone formation rate with a significant increase in osteoblasts [11]. *In vitro* experiments on mouse bone marrow-derived mesenchymal stem cells have also shown that depletion of *Per2* promotes osteogenic differentiation ability [10]. Furthermore, our previous and current studies have demonstrated that PER2 enhances the ameloblast differentiation activity of mouse ameloblast-lineage cells [16] and the odontoblastic/osteogenic differentiation potential of hDPSCs, respectively. These results suggest that PER2 plays multifunctional and complex roles in different cell types. In summary, both *in vitro* and *in vivo* evidence support the notion that PER2 enhances the odontoblastic/osteogenic differentiation potential of hDPSCs.

Increasing evidence indicates that mitochondria participate in regulating cellular functions. Mitochondria play essential roles in stem cell maintenance, proliferation and differentiation [19]. Emerging evidence has demonstrated that oxygen consumption rate and intracellular ATP content are significantly upregulated during differentiation of mesenchymal stem cells (MSCs) to osteoblasts [36]. A recent study has shown that mitochondrial metabolism regulates cementoblastic differentiation of human periodontal ligament stem cells through changes in MMP, ATP synthesis, mitochondrial dynamics, and oxygen consumption rate [37]. Mitochondrial fission–fusion dynamics and bioenergetics, including ATP generation, are strongly influenced by circadian rhythms [22,23]. To explore the correlation between PER2 expression and mitochondrial metabolism during the odontoblastic/osteogenic differentiation of hDPSCs, mitochondrial metabolic status was detected. In the present study, odontoblastic/osteogenic differentiation induction in hDPSCs promoted ATP synthesis, and PER2 enhanced this process. ATP synthesis through oxidative phosphorylation is a fundamental mitochondrial function that is essential to multicellular life [25]. Similarly, PER2 upregulated MMP levels, as evidenced by the accumulation of JC-1 aggregates in PER2-overexpression cells and the reduction in Mitotracker Red CMXRos staining in *PER2*-sh2 cells. MMP provides the driving force for many mitochondrial functions and behaviors, such as the movement of ions and proteins and ATP generation [25]. As both MMP and ATP levels were altered in correlation with PER2 expression, it is believed that PER2 may modulate mitochondrial bioenergetics to accelerate the odontoblastic/osteogenic differentiation of hDPSCs.

The morphology of mitochondria is in dynamic equilibrium that responds to cytoplastic milieu and is constantly undergoing fusion and fission events [38]. Considering the close connection between mitochondrial dynamics and functions, and the involvement of MMP in mitochondrial fusion/fission dynamics [25], it is plausible that mitochondrial fission–fusion dynamics may change during PER2-regulated DPSCs’ differentiation. Mitochondrial fusion is mediated by proteins MFN1, MFN2 and OPA1, while mito-fission is mainly executed by DRP1 and MFF [19]. Assessing the expression levels of fusion and fission proteins provides valuable insights into the dynamic status of mitochondria [39]. Our results showed that overexpression of *PER2* in hDPSCs under odontoblastic/osteogenic induction led to an increase in the expression of the fusion-related protein MFN1 and MFN2, while the expression of fission-related protein MFF and DRP1 decreased. These findings are consistent with previous reports showing a relationship between cell differentiation and mitochondrial dynamics. For instance, MFN2 depletion impaired the differentiation of induced pluripotent stem cells [40], and loss of MFN2/OPA1 in embryonic stem cells hindered their differentiation [41]. Future in-depth research is required to fully elucidate the mitochondrial dynamics and regulation involved in PER2-mediated hDPSCs’ odontoblastic/osteogenic differentiation. Despite the limitations of our results, they suggested that PER2 enhanced the odontoblastic/osteogenic differentiation of hDPSCs through mitochondrial metabolic regulation.

Recent studies in animal disease models have demonstrated the potential of using drugs that regulate circadian genes to treat metabolic dysfunction, cancer, and sleep disorder [29]. In our present study, the circadian clock PER2 plays fundamental roles in promoting odontoblastic/osteogenic differentiation of hDPSCs. The findings highlight the potential of modulating PER2 to enhance dentin regeneration and tissue engineering. Further research using animal models is necessary to explore the effects of PER2 on the reparative dentin formation and to elucidate the underlying mechanism.

## 4. Materials and Methods

### 4.1. Ethics Statement

All experiments using animals followed guidelines approved by the Institutional Animal Care and Use Committee, Sun Yat-sen University (SYSU-IACUC-2022-001948). This study fully complied with the ARRIVE 2.0 protocol. All human tissues were collected at the Hospital of Stomatology, Sun Yat-sen University, with informed consent. The study was approved by the Medical Ethical Committees of the Hospital of Stomatology, Sun Yat-sen University (KQEC-2022-84-02).

### 4.2. Reagents and Antibodies

The reagents used in the study were: ascorbic acid (A4544, Sigma-Aldrich, St. Louis, MI, USA), sodium β-glycerophosphate (G9422, Sigma-Aldrich), dexamethasone (D4902, Sigma-Aldrich), insulin (HY-P0035, MCE, Monmouth Junction, NJ, USA), indomethacin (I7378, Sigma), 3-isobutyl-1-methyl-xanthine (IBMX) (I5879, Sigma), α-MEM (Gibco, Waltham, MA, USA), FBS (Biological Industries, Kibbutz Beit-Haemek, Israel), penicillin–streptomycin (Gibco), GlutaMAX™ (35050061, Gibco), collagenase type I (C0130, Sigma), dispase (58817100, Sigma), polyethylenimine linear (PEI) MW40000 (YEASEN, Shanghai, China), PMSF (ST506, Beyotime, Shanghai, China), RIPA (P0013B, Beyotime, China). Primary antibodies included PER2 (NBP2-24616, Novus Biologicals, Centennial, CO, USA), GAPDH (AF0006, Beyotime, China), β-actin (AF0003, Beyotime, China), DSPP (73632, Santa), DMP1 (NBP1-45525, Novus Biologicals), MFN1 (D6E2S, CST), MFN2 (D1E9, CST), MFF (E5W4M, CST), DRP1 (D8H5, CST).

### 4.3. Dental Pulp Tissue Preparation

Human dental pulp tissues were extracted from healthy third molars (*n* = 3). After tooth extraction, the teeth were washed in PBS and the pulp was separated from the outer layer of hard tissue immediately. The dental pulps were fixed in 4% paraformaldehyde (PFA) overnight, dehydrated in a graded ethanol series, embedded in paraffin, and serially sectioned at 5 μm. The sections were processed for immunohistochemistry and immunofluorescence using the antibodies described below.

### 4.4. Cell Culture

Human impacted third molars were collected from 14- to 24-year-old patients (*n* = 15), with informed consent. Dental pulp tissue was retrieved and digested with 3 mg/mL collagenase type I and 4 mg/mL dispase in α-MEM for 15 min at 37 °C. Cells were maintained in α-MEM supplemented with 20% FBS, 1×GlutaMAX™ and antibiotics (100 U/mL) at 37 °C in 5% CO_2_ humidified atmosphere. Cells at 3–6 passages were used for further experiments, and the concentration of FBS was reduced to 10% for the cell incubation (control medium, CM). The odontoblastic/osteogenic medium (OM) was α-MEM supplemented with 10% FBS,100 U/mL penicillin–streptomycin, 1×GlutaMAX™, 10 mM sodium β-glycerophosphate, 100 nM dexamethasone and 50 μg/mL ascorbic acid. The adipogenic medium (AM) was α-MEM supplemented with 10% FBS,100 U/mL penicillin–streptomycin, 1×GlutaMAX™, 200 μM indomethacin, 0.5 mM IBMX, 10 μg/mL insulin and 1 μM dexamethasone. Each group of cells used in different experiments was specified in Table S1.

### 4.5. Flow Cytometry Analysis

For identification analysis, hDPSCs were identified by the positive expression of MSC surface markers (CD73, CD90 and CD105) and the negative expression of hematopoietic antigens (CD34 and CD45). Human DPSCs at third passage were resuspended as single cell suspensions and incubated with fluorochrome-conjugated anti-human CD34-FITC, CD45-PE-CY5, CD73-FITC, CD90- PE-CY5, and CD105-PE (BD Biosciences, Franklin Lakes, NJ, USA) antibodies at 4 °C for 30 min. The samples were detected and analyzed by flow cytometry (BD Biosciences).

### 4.6. Alkaline Phosphatase, Oil Red O and Alizarin Red S Staining

For alkaline phosphatase (ALP) staining, cells were cultured for 7 days in OΜ medium, fixed in 4% paraformaldehyde and stained with an ALP staining kit according to the manufacturer’s protocol (C3206, Beyotime, China). The formation of mineralized nodules of cells cultured for 21 days in OΜ medium were evaluated by Alizarin Red S staining (ALIR-10001, Cyagen, Taicang, China) and the lipid droplets of cells cultured in AΜ medium for 19 days were stained with Oil-Red O (OILR-10001, Cyagen, China).

### 4.7. PER2 Knockdown and Overexpression in hDPSCs

For *PER2* knockdown, the lentivirus expression vectors *PER2*-sh1, *PER2*-sh2 and control (empty vector) were purchased from GeneChem (Shanghai, China). For *PER2* overexpression (*PER2*-OE), the lentivirus expression vectors pCDNA3.1-*PER2*-3×FLAG and the control vector were purchased from Miaolingbio (Wuhan, China). Control vectors or *PER2* knockdown/overexpression vectors, pMD2G and psPAX2 vectors were co-transfected into 293 T cells with PEI according to the manufacturer’s protocol. After 48 h, the supernatant in 293 T cell dishes was collected for lentivirus. Human DPSCs were transfected with the corresponding lentivirus particles and renamed based on the lentiviral vectors used, and the final efficiency of knockdown or overexpression of *PER2* was evaluated by qRT-PCR and Western blot analysis.

### 4.8. Western Blot

Total proteins of hDPSCs were extracted using RIPA buffer supplemented with 1 mM PMSF. Proteins were subjected to SDS-PAGE and then electrophoretically transferred onto PVDF membranes (Millipore, Burlington, MA, USA). Specific primary antibodies included PER2 (1:1000), DSPP (1:500), DMP1 (1:500), MFN1 (1:1000), MFN2 (1:1000), MFF (1:1000), DRP1 (1:1000), β-actin (1:2000) and GAPDH (1:2000). The immunoreactive proteins were detected by an ECL system (Millipore). GAPDH or β-actin was used as the normalized control for total protein lysis buffer.

### 4.9. Quantitative Real-Time PCR (qRT-PCR)

Total RNA of hDPSCs was extracted using an RNA-Quick purification kit (YISHAN Biotechnology, Shanghai, China) according to the manufacturer’s protocols. Total RNA was reverse transcribed into cDNA using Hifair^®^ Ⅲ 1st Strand cDNA Synthesis SuperMix for qPCR (YEASEN, China). qRT-PCR was performed using Hieff^®^ qPCR SYBR Green Master Mix (YEASEN, China). The relative gene expression was calculated using the equation 2^−Δ(ΔCt)^, where ΔCt = Ct (mRNA) − Ct (GAPDH). PCR primers for each gene are listed in Table S2.

### 4.10. CCK-8 Assay

To examine the impact of *PER2* knockdown or overexpression on the proliferation of hDPSCs, cells were seeded into 96-well plates and cultured for 1, 3, 5, and 8 days. After incubation with Cell Counting Kit-8 (C0037, Beyotime, China), the OD value at 450 nm was measured.

### 4.11. In Vivo Transplantation of hDPSCs

The 4-week-old male BALB/c nude mice were purchased from the Laboratory Animal Center of Sun Yat-sen University (Guangzhou, China) and housed at 22–24 °C and 55–60% humidity under specific pathogen-free (SPF) conditions. Mice had free access to a standard rodent chow diet and water for at least 1 week before the study.

Human DPSCs were transfected with the control lentivirus particles or *PER2*-overexpression lentivirus particles. The control and *PER2*-overexpression hDPSCs (1 × 10^6^) were then suspended and loaded onto the β-tricalcium phosphate (β-TCP) blocks (Biomaterials Engineering Research Center of Sichuan University, China) and the hDPSCs-β-TCP scaffolds were cultured in OM for 24 h (named Con and *PER2*-OE, respectively). After that, the scaffolds were transplanted into 6-week-old male BALB/c nude mice (*n* = 4) by dorsal subcutaneous transplantation. Each group of hDPSCs-β-TCP scaffolds used in mice is specified in Table S3. Grafts were harvested after 8 weeks of transplantation. Transplanted tissues were fixed with 4% formaldehyde overnight, demineralized, dehydrated in a graded ethanol series, embedded in paraffin, and serially sectioned at 5 μm. Sections were analyzed by HE, immunofluorescence and Masson’s trichrome staining.

### 4.12. Histology, Immunohistochemistry and Immunofluorescence

After deparaffinization and rehydration, the samples were stained with hematoxylin–eosin and Masson’s trichrome (G1006, Servicebio, Wuhan, China). The sections were subjected to microscopic analysis. The matrix-like tissue area and collagen volume were measured by Image-Pro Plus 6.0 (Media Cybernetics, Silver Spring, MD, USA).

For immunohistochemistry, the slices were treated with pepsin for 30 min at 37 °C to expose antigens. Immunohistochemistry was performed with a streptavidin-HRP-DAB kit (Cwbio, Suzhou, China). Samples were incubated overnight at 4 °C with the primary antibodies PER2 (1:200). After incubation, the samples were managed according to the manufacturer’s protocols. The sections were then counterstained with hematoxylin. Light yellow to brown staining was recorded as positive immunostaining.

For immunofluorescence staining, paraffin sections were treated with anti-PER2 antibody (1:50) followed by FITC goat anti-rabbit IgG secondary antibody (1:200, E031220, EarthOx, Millbrae, CA, USA) and anti-DSPP antibody (1:50) followed by DyLight 488 AffiniPure goat anti-mouse IgG secondary antibody (1:200, EO32210, EarthOx).

For cell immunofluorescence, cells were fixed with 4% PFA for 20 min, rinsed with PBS, and blocked in serum in a 37 °C incubator for 1 h. Anti-PER2 and anti-DSPP antibodies were used in overnight incubations at 4 °C. Cells were then incubated with secondary antibody as mentioned above for 1 h at room temperature, followed by staining with DAPI. The immunofluorescence paraffin sections and cells were viewed and imaged with a confocal laser scanning microscope.

### 4.13. Measurement of ATP Level

Cells were collected by lysis buffer provided in the ATP assay kit (S0026, Beyotime, China). Briefly, total intracellular ATP of hDPSCs was extracted using ATP lysis buffer, the mixtures were centrifuged, and the supernatants were transferred to a new tube. Intracellular ATP levels were detected by luminometer with a microplate reader. The total protein content was measured by the BCA protein assay kit (Cwbio, China). ATP levels were normalized to the corresponding total protein content.

### 4.14. Mitochondrial Membrane Potential Detection

JC-1 and Mito-Tracker Red CMXRos staining of mitochondria is dependent on the mitochondrial membrane potential. The cells transfected *PER2*-sh2 and the control lentiviral vector exhibited fluorescence upon excitation with a 488 nm laser. However, this laser’s spectral range overlapped with the fluorescence emission spectra of the JC-1 monomer, rendering it unsuitable for detecting MMP in the *PER2*-sh2 and the control cells. As a result, Mito-Tracker Red CMXRos dye was sought for MMP detection in these cells. Mito-Tracker Red CMXRos is suitable for fluorescent double-labeling experiments, and similar to JC-1, the staining of mitochondria with Mito-Tracker Red CMXRos is dependent on MMP. Cells were cultured in OM medium for 3 days and then stained with a JC-1 staining kit (monomer: 490/530; aggregate: 525/590; C2006, Beyotime, China) or Mitotracker Red CMXRos (100 nM, 15 min, 579/599; C1035, Beyotime, China) according to the manufacturer’s protocol. After that, living cells were viewed and imaged with a confocal laser scanning microscope.

### 4.15. Statistical Analysis

All quantitative experiments were performed in triplicate. GraphPad Prism 7.0 software was used for data analyses and graphing. All quantitative data are presented as the mean ± sem. Statistical comparisons between two experimental groups were analyzed by unpaired, two-tailed Student’s *t*-test. Multiple comparison tests were performed using one-way ANOVA for more than two groups.

## 5. Conclusions

In conclusion, the expression of PER2 was increased during the odontoblastic/osteogenic differentiation of hDPSCs. The upregulation of PER2 promoted odontoblastic/osteogenic differentiation, leading to collagen secretion and osteodentine-like structure formation, which was associated with changes in mitochondrial metabolism. This study provides new perspectives in the regulatory mechanisms underlying odontoblastic/osteogenic differentiation in hDPSCs.

## Data Availability

The data used and/or analyzed during the current study are contained within the manuscript or available from the corresponding author on reasonable request.

## Supplementary Materials

The following supporting information can be downloaded at: https://www.mdpi.com/article/10.3390/ijms241310661/s1.
