# Supplementary material for: PER2 Promotes Odontoblastic/Osteogenic Differentiation of Dental Pulp Stem Cells by Modulating Mitochondrial Metabolism
Source: Int J Mol Sci. 2023 Jun 26;24(13):10661. doi: 10.3390/ijms241310661 (PMC10341716; doi:10.3390/ijms241310661)
Supplement: Supplementary file 1 [file ijms-24-10661-s001.zip › ijms-2412264-supplementary.pdf]

PER2 promotes odontoblastic/osteogenic differentiation of dental pulp stem cells by modulating mitochondrial metabolism

Wushuang Huang[1,2,3], Qi Huang[1,2,3], Hongwen He[1,2,3,*], Fang Huang[1,2,3,*]

[1] Hospital of Stomatology, Sun Yat-sen University, Guangzhou, 510055, China

[2] Guangdong Provincial Key Laboratory of Stomatology, Guangzhou, 510055, China

[3] Institute of Stomatology, Sun Yat-sen University, Guangzhou, 510055, China

* Authors to whom correspondence should be addressed.

Supplementary Figures

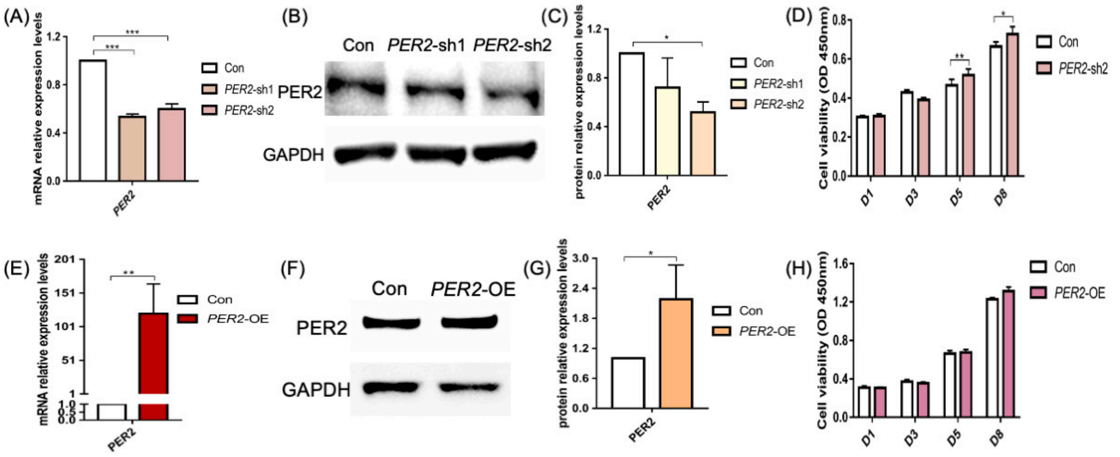

Figure S1. Construction of *PER2*-knockdown and -overexpression hDPSCs and detection of the cell proliferation.

(**A-C**) The knockdown efficiency of PER2 by qRT-PCR (A) and western blot (B, C) analysis, (C) showed quantitative analysis of PER2 protein expression of (B).

(**D**) CCK-8 assay showed that cell proliferation rate of *PER2*-sh2 and the control hDPSCs did not show significant difference cultured in CM for 1 day and 3 days, while cell proliferation rate were slightly higher in *PER2*-sh2 hDPSCs compared with that in the control group cultured in CM for 5 days and 8 days.

(**E-G**) The overexpression efficiency of PER2 by qRT-PCR (E) and western blot (F, G) analysis, (G) showed quantitative analysis of PER2 protein expression of (F).

(**H**) Cell proliferation rate of *PER2*-OE and the control hDPSCs did not show significant difference cultured in CM for 1 day, 3 days, 5 days and 8 days.

Data are presented as mean±SEM. *P < 0.05, **P < 0.01, ***P < 0.001

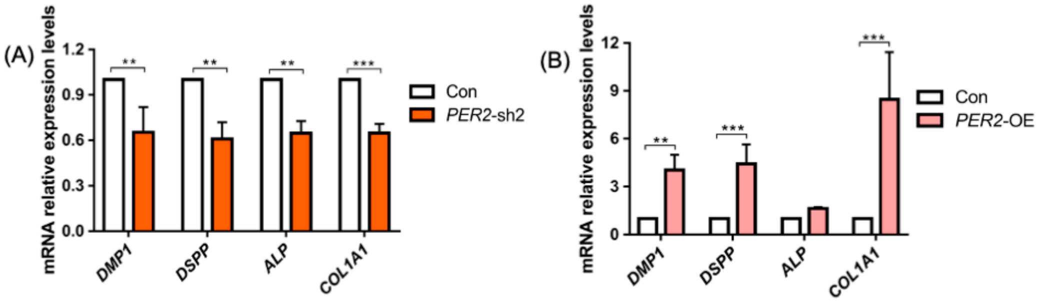

Figure S2. qRT-PCR analysis of expression of differentiated-related markers.

(**A, B**) Cells were cultured in OM for 7 days and harvested for qRT-PCR. *PER2* depletion caused reductions in *DMP1*, *DSPP*, *ALP*, *COL1A1* mRNA expression with statistical significance (A); *PER2*-overexpression up-regulated mRNA expression of *DMP1*, *DSPP*, *COL1A1* with statistical significance, while *ALP* mRNA expression was only slightly increased in *PER2*-OE hDPSCs.

Data are presented as mean±SEM. **P < 0.01, ***P < 0.001

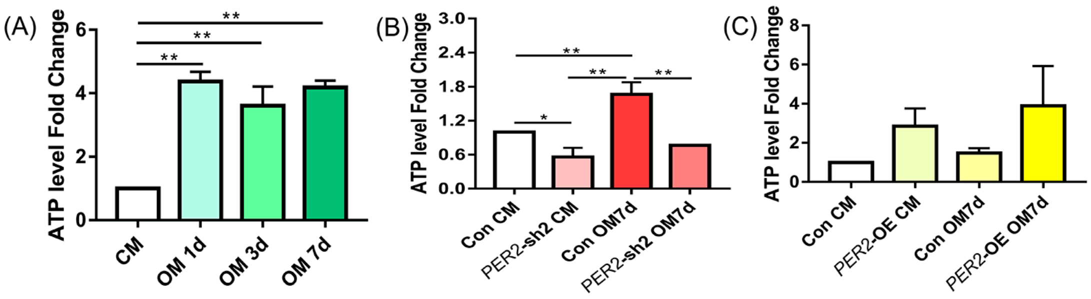

Figure S3. Intracellular ATP generation in hDPSCs under different cultured conditions.

(**A**) hDPSCs were cultured in CM or OM and harvested on 1, 3, 7 days respectively. Compared with cultured in CM, more intracellular ATP was detected in the hDPSCs incubated in OM, while the amount of ATP generation did not show statistic difference among the three group of hDPSCs cultured in OM.

(**B**) Cells were cultured in CM or OM for 7 days. Less intracellular ATP was measured in *PER2*-sh2 hDPSCs compared with that in the control group, whether the hDPSCs cultured in CM or OM.

(**C**) Cells were cultured in CM or OM for 7 days. Overexpression of *PER2* promoted intracellular ATP generation though without statistical significance.

Data are presented as mean±SEM. *P < 0.05, **P < 0.01, ***P < 0.001

Supplementary Tables

Table S1. Cells cultured in different groups

Cells groups	Cultured conditions	Experiments
hDPSCs	Cells cultured in CM or OM for 7 days and 21 days respectively, and in AM for 19 days	immunofluorescence; Alizarin red S staining; Oil Red O staining.
hDPSCs-Con; hDPSCs-*PER2*-sh1; hDPSCs-*PER2*-sh2	Cells cultured in CM for 3 days	Knockdown efficiency detected by qRT-PCR and western blot
hDPSCs-Con; hDPSCs-*PER2*-OE	Cells cultured in CM for 3 days	Overexpression efficiency detected by qRT-PCR and western blot
hDPSCs-Con; hDPSCs-*PER2*-sh2	Cells cultured in CM for 1, 3, 5, 8 days	CCK-8 assay
hDPSCs-Con; hDPSCs-*PER2*-OE	Cells cultured in CM for 1, 3, 5, 8 days	CCK-8 assay
hDPSCs-Con; hDPSCs-*PER2*-sh2	Cells cultured in OM for 7 days or 21 days	qRT-PCR, western blot, ALP staining, Alizarin red S staining
hDPSCs-Con; hDPSCs-*PER2*-OE	Cells cultured in OM for 7 days or 21 days	qRT-PCR, western blot, ALP staining, Alizarin red S staining,
hDPSCs	Cells cultured in CM for 7 days or OM for 1, 3, 7 days	ATP detection
hDPSCs-Con; hDPSCs-*PER2*-sh2	Cells cultured in CM or OM for 7 days	ATP detection
hDPSCs-Con; hDPSCs-*PER2*-OE	Cells cultured in CM or OM for 7 days	ATP detection
hDPSCs-Con; hDPSCs-*PER2*-sh2	Cells cultured in OM for 3 days	Mitotracker Red CMXRos staining
hDPSCs-Con; hDPSCs-*PER2*-OE	Cells cultured in OM for 3 days	JC-1 staining

Table S2. List of primers for qRT-PCR

Gene	Forward primers (5’-3’)	Reverse primers (3’-5’)
PER2	AGATGTGGACATGAGCAGTGG	TGGGTTGTGTTCAGATTTTGCC
COL1A1	GTGCGATGACGTGATCTGTGA	CGGTGGTTTCTTGGTCGGT
ALP	AACATCAGGGACATTGACGTG	GTATCTCGGTTTGAAGCTCTTCC
DMP1	CACTCAAGATTCAGGTGGCAG	TCTGAGATGCGAGACTTCCTAAA
DSPP	GCATTTGGGCAGTAGCATGG	CACTGGCATTTAACTCATCCTGT

Table S3. hDPSCs-β-TCP scaffolds used in the mice

Mice	Groups	Experiments
6-week-old male BALB/c nude mice (n=4)	Con: β-TCP blocks loaded with control hDPSCs were transplanted into mice by left dorsal subcutaneous transplantation for 8 weeks.<br>*PER2*-OE: β-TCP blocks loaded with *PER2*-overexpression hDPSCs were transplanted into mice by right dorsal subcutaneous transplantation for 8 weeks.	H&E staning;<br>Masson’s trichrome staining;<br>immunofluorescence
